# AD‐related plasma biomarkers in centenarians: links to cognition and neuropathology

**DOI:** 10.1002/alz.70969

**Published:** 2025-12-19

**Authors:** Linda M. C. Lorenz, Susan K. Rohde, Maruelle C. Luimes, Annemieke J. M. Rozemuller, Netherlands Brain Bank, Marc Hulsman, Marieke J. I. Graat, Myke E. van der Hoorn, Dominique A. H. Daatselaar, Mariam Gouda, Jeroen J. M. Hoozemans, Sietske A. M. Sikkes, Charlotte E. Teunissen, Henne Holstege

**Affiliations:** ^1^ Genomics of Neurodegenerative Diseases and Aging, Department of Human Genetics Vrije Universiteit Amsterdam, Amsterdam UMC Amsterdam the Netherlands; ^2^ Alzheimer Center Amsterdam, Department of Neurology Vrije Universiteit Amsterdam Amsterdam the Netherlands; ^3^ Amsterdam Neuroscience Neurodegeneration Research Program, Amsterdam UMC Amsterdam the Netherlands; ^4^ Department of Pathology Amsterdam Neuroscience, Amsterdam UMC Amsterdam the Netherlands; ^5^ Netherlands Institute for Neuroscience Amsterdam the Netherlands; ^6^ Neurochemistry Laboratory, Department of Clinical Chemistry Vrije Universiteit Amsterdam, Amsterdam UMC Amsterdam the Netherlands; ^7^ Faculty of Behavioural and Movement Sciences, Department of Clinical Developmental Psychology and Clinical Neuropsychology Vrije Universiteit Amsterdam Amsterdam the Netherlands; ^8^ VIB‐KU Leuven: Center for Brain & Disease Research Leuven Belgium

**Keywords:** aging, amyloid‐beta, blood‐based biomarkers, centenarians, cognitive performance, glial fibrillary acidic protein, neurofilament light, phosphorylated tau 181, *post mortem* neuropathology, tau

## Abstract

**INTRODUCTION:**

Whether Alzheimer's disease (AD)‐associated plasma biomarkers reflect cognitive performance and neuropathology in the oldest old remains unclear.

**METHODS:**

In plasma samples from 255 centenarians from the longitudinal 100‐plus Study (median age 101.2 years), we quantified biomarkers amyloid beta (Aβ)42/40 ratio, Aβ40, Aβ42, phosphorylated tau 181 (pTau‐181)/Aβ42 ratio, pTau‐181, neurofilament light (NfL), and glial fibrillary acidic protein (GFAP) concentrations. These were associated with same‐day measures of cognitive performance and, for centenarians who donated their brain (*n* = 60), with *post mortem* Aβ and tau neuropathology.

**RESULTS:**

Cognition ranged from high to early cognitive decline (median Mini‐Mental State Examination [MMSE] score = 26). Lower plasma Aβ40 and Aβ42 are associated with poorer executive functioning, attention/processing speed, and higher Aβ neuropathology. Elevated plasma NfL and GFAP are associated with poorer executive functioning, slower processing speed, and Aβ and tau neuropathology. Higher plasma pTau‐181 and the pTau‐181/Aβ42 ratio are associated with Aβ and tau neuropathology, but not with cognitive performance. The Aβ42/40 ratio was uninformative.

**DISCUSSION:**

Plasma Aβ, NfL, and GFAP detected neuropathology and early cognitive decline in centenarians; plasma pTau‐181 and the pTau‐181/Aβ42 ratio primarily report more advanced neuropathology.

**Highlights:**

- Lower plasma Aβ40 and Aβ42 concentrations are associated with poorer executive functioning and higher Aβ neuropathology, and thus may detect early cognitive decline in centenarians.
- Higher plasma pTau‐181 concentrations and the pTau‐181/Aβ42 ratio are strongly associated with Aβ and tau neuropathology; however, they are not associated with cognitive performance.
- Higher NfL concentrations are associated with higher Aβ and tau neuropathology.
- Higher plasma NfL and GFAP concentrations are associated with poorer attention and processing speed and may detect early cognitive decline in centenarians.

## 1 BACKGROUND

Alzheimer's disease (AD) is the most prevalent cause of dementia and affects more than 50 million individuals worldwide, a number projected to exceed 150 million by 2050.^1^ AD is characterized by cognitive decline, neurodegeneration, and neuroinflammation. Traditionally, AD is confirmed after death by three hallmarks of AD neuropathologic change (ADNC), including extracellular amyloid beta (Aβ) plaques, intracellular neurofibrillary tau tangles (NFTs), and neuritic plaques (NPs).^2^ However, the development of blood‐based biomarkers that reflect underlying neuropathology has strongly improved in vivo differentiation between individuals with and without underlying amyloid pathology.^3^, ^4^

Among the panel of validated AD‐associated blood‐based biomarkers, lower concentrations of soluble isoforms of Aβ40 and Aβ42 constitute a key change in the plasma of AD patients. The decreased concentration of soluble Aβ42, in particular, is thought to reflect its sequestration and aggregation in the brain.^5^ As amyloid accumulation has been shown to initiate tau hyperphosphorylation and spreading, it follows that increased plasma phosphorylated tau‐181 (pTau‐181) and elevated tau‐positron emission tomography (PET) signals^6^, ^7^ are not only associated with Braak NFT staging but also correlate strongly with the burden of Aβ neuropathology.^7^, ^8^, ^9^, ^10^ In addition, increased plasma concentrations of blood‐based neurofilament light (NfL) reflect axonal degeneration,^11^ and increased plasma concentrations of glial fibrillary acidic protein (GFAP) reflect astrocyte activation in response to neuroinflammation, and are associated with increased Aβ neuropathology.^12^, ^13^

Previous studies associated in vivo blood‐based biomarkers with *post mortem* neuropathological substrates.^14^, ^15^, ^16^, ^17^ These studies mainly involved patients with mild cognitive impairment (MCI), AD, or other dementias within the age range of 50 to 85 years. However, AD‐related neuropathology has been observed to increase with age, even in cognitively unimpaired individuals,^18^, ^19^, ^20^ and previous reports indicated that, in parallel, AD‐associated blood‐based biomarker concentrations also increase with age.^21^ This led us to question to what extent AD‐associated blood‐based biomarker concentrations in the oldest old reflect cognitive status and age‐related neuropathological changes associated with AD. We therefore analyzed plasma levels of the Aβ42/40 ratio, Aβ40, Aβ42, the pTau‐181/Aβ42 ratio, pTau‐181, NfL, and GFAP in a unique cohort of centenarians with cognitive abilities ranging from high‐performing to having initial symptoms of cognitive decline, of whom a subset agreed to *post mortem* brain donation. Uniquely, this allowed us to explore associations with (1) cognitive performance and (2) AD‐related *post mortem* neuropathology.

## 2 METHODS

### 2.1 Sample

The 100‐plus Study is an ongoing longitudinal cohort study that recruits centenarians from across the Netherlands, who self‐report to be cognitively healthy, with confirmation by proxy, and who agree to neuropsychological testing and blood sampling. The study includes the collection of multi‐domain data during home visits. Detailed descriptions of the overall study design, collection procedures, and cohort characteristics were published previously^22^ and are available in Supplementary Methods. For the present study, we included 255 centenarians for whom plasma samples were available for biomarker analysis. *Post mortem* brain tissue was available for 60 centenarians from this group (Figure S1). The study was approved by the Amsterdam UMC Medical Ethics Committee (registration 2013.82 and 2016.440). All participants provided written informed consent, and data were collected in accordance with the Declaration of Helsinki.

### 2.2 Demographic data and clinical assessments

Demographic information (age, sex, educational attainment) and physical functioning, assessed using the Dutch version of the Barthel Index (BI) questionnaire for activities of daily living (ADL),^23^ were collected (see Supplementary Methods and prior documentation).^22^

### 2.3 Blood collection

Ethylenediaminetetraacetic acid (EDTA) K_2_ non‐fasted blood samples were obtained via venipuncture using a 20G or 21G needle into 6 mL tubes, in accordance with established protocols.^22^ All plasma samples were randomized across assay batches. For details, see Supplementary Methods.

### 2.4 Single molecule array (Simoa) plasma biomarker measurements

Plasma pTau‐181 was analyzed using the Simoa Advantage Kit version 2.0. Plasma samples Aβ40, Aβ42, NfL, and GFAP were analyzed simultaneously with the Neurology 4‐Plex‐E Advantage (N4PE) Kit on a Simoa HD‐X analyzer (Quanterix, Billerica, USA) by the Neurochemistry lab Amsterdam.

**RESEARCH‐IN‐CONTEXT**

1. **Systematic review**: The authors conducted a literature review using traditional database searches. Prior studies primarily investigated the association between blood‐based biomarkers, cognitive performance, and AD‐associated neuropathology in younger elderly populations or in patients with neurodegenerative disorders. Limited data are available on such associations in non‐demented centenarians.
2. **Interpretation**: Our results demonstrate that blood‐based biomarkers (plasma Aβ, NfL, and GFAP) may detect early cognitive decline in centenarians. Notably, plasma pTau‐181 and the pTau‐181/Aβ42 ratio showed a strong association with *post mortem* AD neuropathology, but not with cognitive performance. This dissociation suggests that more substantial cognitive impairment may be necessary for plasma pTau‐181 concentrations and the pTau‐181/Aβ42 ratio to reach levels detectable in relation to cognitive performance. These findings imply a potential disconnect between neuropathology and its clinical manifestation in this resilient population.
3. **Future directions**: Future studies should aim to replicate these findings in larger cohorts of the oldest old. The biomarker panel should be expanded to include additional plasma markers such as pTau‐231 and pTau‐217, as well as markers of age‐related comorbidities, including limbic‐predominant age‐related TDP‐43 encephalopathy (LATE) and vascular neuropathology (e.g., infarcts, atherosclerosis). Furthermore, determinants that distinguish between brain amyloid subtypes, such as diffuse plaques, cored plaques, and cerebral amyloid angiopathy (CAA), may enhance the pathological specificity of blood‐based diagnostics.

### 2.5 Quality control of plasma biomarkers

All measured biomarker concentrations were above the lower limit of quantification (LLOQ), ensuring reliable quantification. For pTau‐181, intra‐assay and inter‐assay variability were maintained within acceptable limits, with coefficient of variation (CV) percentages of 6.1% and 9.2% for low quality control (QC) and 11.4% for high QC, respectively. Quality control procedures included the analyses of two control samples per biomarker on each plate, at both the start and end, achieving a CV percentage below 20%, indicating consistent assay performance. Given GFAP's sensitivity to multiple freeze‐thaw cycles, data from three participants with three freeze‐thaw cycles were excluded to preserve integrity.

### 2.6 Cognitive assessment

Cognitive performance was evaluated with an 11‐test neuropsychological battery assessing cognitive domains: memory, verbal fluency, executive function, visuospatial function, and attention/processing speed, together with the Mini‐Mental State Examination (MMSE)^24^ for global cognitive functioning (see Supplementary Methods and previous publication; Table S1).^22^ Missing scores caused by sensory or motor impairments, fatigue, or test battery adaptations, were imputed using Multiple Imputation by Chained Equations (MICE) version 3.13.0.^25^ Predictive mean matching was selected for its ability to handle non‐normal data distributions, and 74 iterations were performed,^26^ consistent with previous publications of the same cohort.^20^, ^27^ As the amount of imputed data varied per test, the statistical reliability of the imputation was validated by calculating the fraction of missing information (FMI)^28^ and relative increase in variance (RIV) for each model (Table S1). The RIV, which represents the proportional increase in the variance of parameter estimates due to missing data, ranged from 0.021 to 0.653 (mean = 0.202). Although no definitive threshold exists, the RIV mean is below 0.5, a commonly accepted benchmark indicating that the imputation introduced a manageable level of statistical uncertainty.^28^, ^29^ The FMI, which quantifies the overall uncertainty attributable to missing data, ranged from 0.020 to 0.403 (mean = 0.160), a range classified as modest to moderate.^30^ These low mean values confirm the robustness of the imputation procedure, with the mean FMI indicating that, on average, 84% of the information contributing to the final statistical estimates was derived directly from the observed data. The final imputed dataset used for cognitive analyses comprised all 255 centenarians, with the exception of the MMSE, for which the sample was restricted to *n* = 249 centenarians after excluding individuals with ≥6 missing items (Supplementary Methods and Table S2.

### 2.7 *Post mortem* brain tissue and neuropathological evaluation

Among the 255 centenarians with blood samples available, 60 centenarians (24%) donated their brain. Brain autopsies were conducted between 2013 and 2022 by the Netherlands Brain Bank (NBB; Amsterdam, the Netherlands) under the approval of the Amsterdam UMC Ethics Committee (registration 2009.148). Donors provided informed consent for autopsy, tissue storage, and use of pseudonymized clinical and neuropathological data. *Post mortem* brain donation occurred within 5.9 h (interquartile range [IQR]: 5.2 to 6.8), followed by fixation in 10% formalin for ∼4 weeks. A single neuropathologist (A.J.M.R.) performed tissue dissection and diagnosis per Brain Net Europe II and National Institute on Aging and Alzheimer's Association (NIA‐AA) guidelines.^3^, ^31^

### 2.8 Immunohistochemistry and neuropathological assessment

AD neuropathology was assessed using the (1) Thal Aβ phase for the spatiotemporal spread of Aβ plaques (0 to 5),^32^ (2) Braak NFT stage for the spatiotemporal spread of tau tangles (0 to VI),^33^ (3) Consortium to Establish a Registry for Alzheimer's Disease (CERAD) NP score for the frequency of NPs (0 to 3).^34^ These three scores were combined into an (4) ADNC score (0 to 3) according to the criteria of the NIA‐AA.^2^, ^35^ Moreover, we evaluated (5) TAR DNA‐binding protein 43 (TDP‐43 stage) (0 to 3)^36^, presence or absence of (6) hippocampal sclerosis, and (7) the Braak Lewy body (LB) stage (0 to 6).^37^ Further assessments included evaluating (8) global levels of macroscopic atrophy (0 to 3), (9) cerebral atherosclerosis (0 to 3), (10) the presence or absence of cerebral infarcts, and (11) cerebral amyloid angiopathy (CAA) stage (0 to 3)^38^ (see Supplementary Methods for details on neuropathological evaluation and Figure S2).

### 2.9 Quantitative neuropathology of Aβ and tau

The quantitative loads of Aβ and tau neuropathology were determined as the immunopositive percentage of the temporal pole cortex (Brodmann area 38), as described previously.^27^ In short, immunohistochemical staining was conducted for total Aβ (6F/3D; aa [amino acid] 8‐17), and the Aβ40 (MABN11; aa 33‐40) and Aβ42 isoforms (MABN12; aa 35‐42; Table S3). For tau neuropathology, AT8 (pTau‐202/205) predominantly stains pre‐tangles, pTau‐217 stains intermediate to mature tangles, and GT‐38 stains mature to ghost tangles (Figure S3).^39^ As neuropathological work‐up is still in progress, quantitative Aβ and tau measurements are currently unavailable for seven brain donors.

### 2.10 Statistical analysis

For comparative analyses, all test scores, neuropathological scores, and plasma biomarkers were *z*‐standardized to ensure the comparability of effect sizes (*β*), allowing them to be interpreted as the change in the outcome for each 1 standard deviation (SD) change in a given predictor. Robust linear regression models were computed using the MASS package (version 7.3‐60) to reduce outlier impact and address non‐normal residuals. To account for potential heteroscedasticity in the residuals, robust standard errors were calculated using the heteroscedasticity correction (HC2) estimator, which adjusts the variance‐covariance matrix of the model's coefficients to provide more reliable standard error estimates. Estimated *p* values were obtained through the *f.robftest* function from the sfsmisc package (version 1.1.19). For cognitive outcomes, plasma biomarkers were used as independent variables and cognitive test scores as dependent variables. Separate models per plasma biomarker were adjusted for age at blood collection, sex, and educational attainment. For neuropathological outcomes, plasma biomarkers were used as independent variables and neuropathological measures as dependent variables. Separate models per plasma biomarker were adjusted for age at blood collection, sex, the time interval between blood sampling and brain donation, and co‐pathologies (TDP‐43 stage, Braak LB stage, atherosclerosis, cerebral atrophy, hippocampal sclerosis, and the presence of infarcts). The effective number of independent tests (M_eff_) was estimated from biomarker‐by‐outcome correlation matrices with the Li & Ji method using the poolr package (version 1.1‐1). All *p* values were subsequently pooled and adjusted for false discovery rates (FDR) using the Benjamini–Hochberg method and scaled by M_eff_. Two‐sided tests were used throughout, and for this exploratory analysis, a threshold of *Adj.P* < 0.10 was considered statistically significant. Statistical analyses and visualizations were performed using RStudio (version 4.4.2, “Pile of Leaves”).

## 3 RESULTS

### 3.1 Sample characteristics

The study cohort included 255 centenarians (173 females, 82 males) for whom blood samples were available. The median age at cognitive assessment was 101.2 years (IQR 100.3 to 102.2), females had a slightly higher median age at 101.3 years compared to males at 101.1 years (*p *= 0.026; Table 1). The median years of education was 10.0 years (IQR 7.0 to 12.0). Males had higher physical ADL function as measured by BI scores (median: 17.9 vs 14.0, *p* < 0.001) and higher cognitive function as measured by MMSE scores (median: 26 vs 25, *p* = 0.014) than females.

**TABLE 1:** Demographic characteristics and cognitive test scores.

	Total cohort	Median (IQR)/*n* (%) [range]	Females	Median (IQR)/*n* (%) [range]	Males	Median (IQR)/*n* (%) [range]	*p* value
Demographic characteristics							
Age at cognitive assessment (*y*)	*255*	101.2 (100.3 to 102.2) [100.0 to 107.7]	173	101.3 (100.4 to 102.5) [100.0 to 107.7]	82	101.1 (100.3 to 102.2) [100.1 to 106.5]	0.026
Education (y)	*255*	10.0 (7.0 to 12.0) [4.0 to 27.0]	173	9.0 (7.0 to 12.0) [4.0 to 27.0]	82	10.0 (8.0 to 13.0) [5.0 to 20.0]	0.091
Barthel Index (ADL) (range: 0 to 20)	*255*	15.0 (12.0 to 18.0) [0.0 to 20.0]	173	14.0 (11.0 to 17.0) [0.0 to 20.0]	82	17.9 (15.0 to 19.0) [4.0 to 20.0]	**<0.001**
Cognitive test scores							
Visual Association Test trials 1+2 (range: 0 to 12)	*255*	9.0 (5.0 to 11.0) [0.0 to 12.0]	173	9.0 (4.0 to 11.0) [0.0 to 12.0]	82	10.0 (7.0 to 11.0) [0.0 to 12.0]	0.068
CERAD 10 WT reproduction (range: 0 to 30)	*255*	14.2 (11.0 to 17.0) [2.0 to 24.0]	173	14.7 (11.0 to 17.2) [2.0 to 24.0]	82	14.0 (11.0 to 17.0) [4.0 to 23.0]	0.500
CERAD 10 WT delayed (recall) (range: 0 to 10)	*255*	3.00 (1.08 to 5.00) [0.00 to 10.00]	173	3.00 (1.00 to 5.00) [0.00 to 10.00]	82	3.00 (2.00 to 4.80) [0.00 to 9.00]	0.600
Letter fluency (D‐A‐T, 1 min) (total word count)	*255*	22 (16 to 29) [2 to 59]	173	22 (16 to 29) [4 to 59]	82	22 (15 to 32) [2 to 52]	0.600
Animal fluency (1 min), (total word count)	*255*	12.0 (9.0 to 16.0) [2.0 to 30.0]	173	11.0 (8.0 to 14.7) [3.0 to 30.0]	82	13.0 (10.0 to 16.0) [2.0 to 24.0]	**0.009**
Digit span (forward) (range: 0 to 16)	*255*	7.00 (6.00 to 8.00) [4.00 to 11.00]	173	7.00 (5.80 to 8.00) [4.00 to 11.00]	82	8.00 (6.00 to 9.00) [4.00 to 11.00]	**<0.001**
Digit span (backward) (range: 0 to 16)	*255*	5.00 (4.00 to 6.00) [1.00 to 8.00]	173	5.00 (3.76 to 5.76) [1.00 to 8.00]	82	5.00 (4.00 to 6.00) [2.00 to 8.00]	0.100
Key search profile score (range: 0 to 4)	*255*	1.00 (0.60 to 2.00) [0.00 to 4.00]	173	1.00 (0.48 to 2.00) [0.00 to 4.00]	82	2.00 (1.00 to 3.00) [0.00 to 4.00]	**<0.001**
Clock drawing test (range: 0 to 5)	*255*	3.00 (2.68 to 5.00) [0.00 to 5.00]	173	3.00 (2.24 to 5.00) [0.00 to 5.00]	82	3.00 (3.00 to 5.00) [1.52 to 5.00]	0.200
Trail making test A (s)	*255*	120 (86 to 207) [35 to 584]	173	120 (86 to 212) [35 to 485]	82	108 (88 to 191) [48 to 584]	0.600
Trail making test B (s)	*255*	355 (265 to 496) [78 to 920]	173	355 (277 to 500) [94 to 761]	82	356 (250 to 471) [78 to 920]	0.400
MMSE score (range: 0 to 30)	*249*	26.0 (22.0 to 28.0) [12.0 to 30.0]	169	25.0 (22.0 to 27.8) [12.0 to 30.0]	80	26.0 (24.6 to 28.0) [17.0 to 30.0]	**0.014**

Abbreviations: ADL, activities of daily living; CERAD, Consortium to Establish a Registry for Alzheimer's Disease; IQR, interquartile range; MMSE, Mini‐Mental State Examination; WT: word list.

A subset of 60 participants donated their brain after death, with a median interval of 16.1 months (IQR 3.9 to 28.4) [0.1 to 61.4] between blood collection and death. Compared to non‐donors (*n* = 195), donors were slightly older at blood collection (median:102.2 vs 101.3 years, *p* = 0.002) and age at death (median: 103.7 vs 103.0, *p* = 0.010) and had a slightly higher MMSE score (median: 26 vs 25, *p* = 0.006). However, the characteristics of the centenarians in this subset reflected the characteristics of the full cohort, as they did not significantly differ in sex distribution or years of education (both *p* > 0.3). Notably, as most brain donors were recruited early in the study, a modest recruitment‐timing bias is present. Demographic characteristics and cognitive test scores are summarized in Table 1, plasma biomarker concentrations and neuropathology metrics are summarized in Table 2.

**TABLE 2:** Plasma biomarker concentrations and neuropathological substrates.

	Total cohort	Median (IQR) / *n* [range]
Plasma biomarkers		
Age at blood collection (y)	*255*	101.3 (100.3 to 102.4) [100.0 to 107.7]
Aβ42/40 ratio	*255*	0.053 (0.047 to 0.058) [0.017 to 0.087]
Aβ40	*255*	155.20 (129.60 to 191.70) [68.60 to 299.20]
Aβ42	*255*	8.07 (6.51 to 9.85) [2.11 to 17.66]
pTau‐181/Aβ42 ratio	*255*	0.534 (0.388 to 0.741) [0.161 to 2.459]
pTau‐181	*255*	4.05 (3.10 to 5.62) [1.46 to 12.75]
NfL	*255*	50.54 (37.07 to 73.33) [11.87 to 378.65]
GFAP	*252*	251.75 (188.51 to 325.77) [89.04 to 1085.09]
Neuropathology substrates		
Age at brain donation (y)	*60*	103.7 (102.43 to 106.03) [100.54 to 110.30]
Time between brain donation and blood collection (m)	*60*	16.1 (3.9 to 28.4) [0.1 to 61.4]
Thal Aβ phase (range: 0 to 5)	*60*	2 (1 to 4) [0 to 5]
Braak‐NFT stage (range: 0 to VI)	*60*	III (III to IV) [II to V]
CERAD‐NP score (range: 0 to 3)	*60*	1 (0 to 1) [0 to 3]
ADNC (range: none/low/intermediate/high)	*60*	7/27/22/4
Quantitative cortical total Aβ load (%)	*53*	0.56 (0.00 to 1.90) [0.00 to 9.02]
Quantitative cortical Aβ40 load (%)	*55*	0.40 (0.01 to 1.42) [0.00 to 5.53]
Quantitative cortical Aβ42 load (%)	*55*	2.37 (0.28 to 5.16) [0.02 to 11.46]
Quantitative cortical AT8 (pTau‐202/205) load (%)	*55*	0.97 (0.26 to 3.59) [0.00 to 49.19]
Quantitative cortical pTau‐217 load (%)	*55*	0.26 (0.12 to 0.52) [0.02 to 1.85]
Quantitative cortical GT‐38 load (%)	*55*	0.05 (0.03 to 0.14) [0.01 to 0.82]
Thal CAA stage (range: 0 to 2)	*60*	1 (0 to 1) [0 to 2]
TDP‐43 stage (range: 0 to 3)	*59*	0 (0 to 2) [0 to 3]
Atherosclerosis (range: none/mild/moderate/severe)	*60*	14/25/21/0
Cerebral infarcts (no infarcts/infarcts)	*60*	23/37
Braak‐LB stage (range: 0 to 6)	*60*	0 (0–1) [0 to 5]
Hippocampal sclerosis (yes/no)	*60*	14/46
Cerebral atrophy (range: none/mild/moderate/severe)	*60*	32/22/6/0

Abbreviations: ADNC, Alzheimer's Disease Neuropathologic Change; AT8, phosphorylated tau at positions 202/205; Aβ, amyloid beta; CAA, cerebral amyloid angiopathy; CERAD, Consortium to Establish a Registry for Alzheimer's Disease; GFAP, glial fibrillary acidic protein; GT‐38, monoclonal antibody specific for a mature form of tau protein; IQR, interquartile range; LB, Lewy body; m, months; NfL, neurofilament light; NFT, neurofibrillary tangle; NP, neuritic plaque; pTau, phosphorylated tau; TDP‐43, TAR DNA‐binding protein 43. All plasma biomarker concentrations are listed as median (IQR) in pg/mL, with the exception of the Aβ42/40 ratio and the pTau‐181/Aβ42 ratio.

For 85% of the centenarians (*n* = 217), cognitive testing and blood collection occurred during the same study visit, while for 15% of the centenarians (*n* = 38), blood samples were collected at a following study visit, with a median time interval of 3.1 months (IQR: 1.7 to 5.3) [0.2 to 45.5]. The latter occurred because blood collection for the purposes of biomarker analyses was introduced while the study was running, such that we had to revisit several centenarians for blood sample collection.

### 3.2 In vivo plasma biomarkers associate with cognitive performance

**FIGURE 1:**
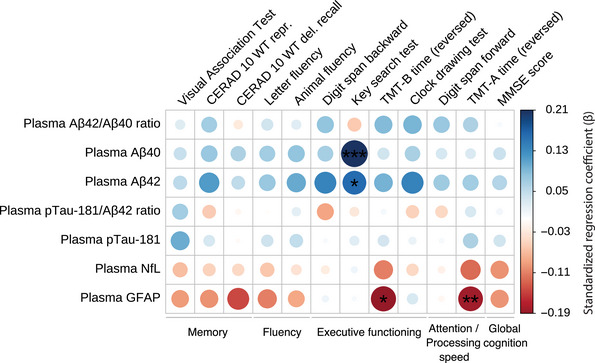
Associations between plasma biomarker concentrations and cognitive tests at time of blood collection. Robust linear regression analyses between plasma biomarkers (rows: amyloid beta (Aβ)42/40 ratio; Aβ42; Aβ40; phosphorylated tau‐181 (pTau‐181)/Aβ42 ratio; pTau‐181; neurofilament light (NfL); glial fibrillary acidic protein (GFAP)) and cognitive performance were conducted across the following tests (columns): Visual Association Test (VAT), CERAD 10 Word Test reproduction, CERAD 10 Word Test delayed recall, letter fluency (D‐A‐T), animal fluency, digit span backwards, Key Search Test, Trail Making Test B (TMT‐B) time (reversed), Clock Drawing Test, digit span forward, Trail Making Test A (TMT‐A) time (reversed), and Mini‐Mental State Examination (MMSE) score. The color scale on the right represents the regression coefficient (*β*), ranging from −0.19 (negative correlation, red) to 0.21 (positive correlation, blue), with larger circles indicating a stronger magnitude of correlation. All variables were standardized (z‐scored) to ensure comparability between the regression coefficients. Robust linear regression models were employed to calculate models. Models were corrected for age at blood collection, sex, and years of educational attainment. *p* values (*≤0.10, **≤0.05, ***≤0.01), were adjusted for false discovery rate (FDR) using the Benjamini–Hochberg method and scaled by the effective number of independent tests (M_eff_) estimated via the Li & Ji method; significance was set at Adj. *p* < 0.10. *n* = 243–255. Numerical regression coefficients and *p*‐values can be found in Table S4.

**FIGURE 2:**
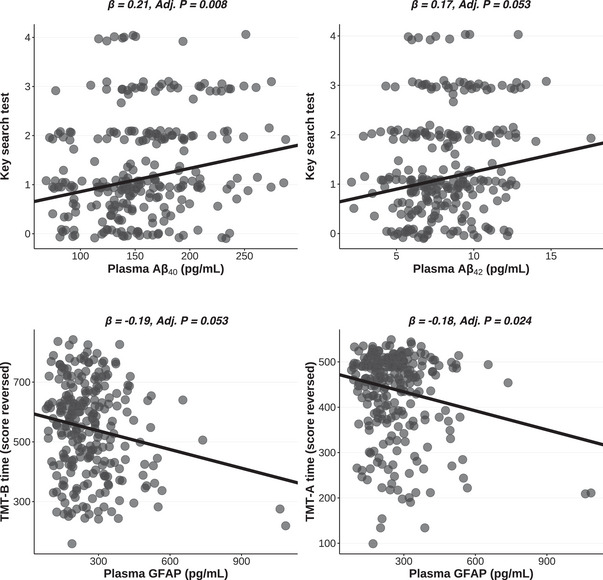
Scatterplots of associations between plasma biomarkers and cognitive tests. Each panel presents a robust linear regression fit (solid line) along with the standardized regression coefficient (*β*) and *p* value for the predictor variable (plasma biomarker). *n* = 243 to 255.

Lower concentrations of Aβ40 and Aβ42, along with higher concentrations of NfL and GFAP, were modestly yet consistently correlated with poorer cognitive performance (Figure 1 and Table S4). In contrast, the plasma Aβ42/40 ratio, pTau‐181, and pTau‐181/Aβ42 ratio did not correlate with cognitive performance. Specifically, we observed significant associations with executive functioning, a domain that involves planning and prioritization. Higher plasma Aβ40 and Aβ42 were significantly associated with better performance on the Key Search Test (*β =* 0.21, *Adj.P* = 0.008; *β =* 0.17, *Adj.P* = 0.053, respectively). Moreover, for attention/processing speed, higher plasma GFAP was associated with poorer performance, reflected by an approximately 18‐s slower completion time on the Trail Making Test A (TMT‐A) for each 1 SD increase (*β =* ‐0.18, *Adj.P* = 0.024). Similarly, higher plasma GFAP was associated with poorer performance on the Trail Making Test B (TMT‐B), with an approximately 30‐s slower completion time for each 1 SD increase (*β =* ‐0.19, *Adj.P* = 0.053; Figure 2). Raw cognitive test distributions are shown in Figure S4.

### 3.3 In vivo plasma biomarkers associate with *post mortem* AD‐associated neuropathology

**FIGURE 3:**
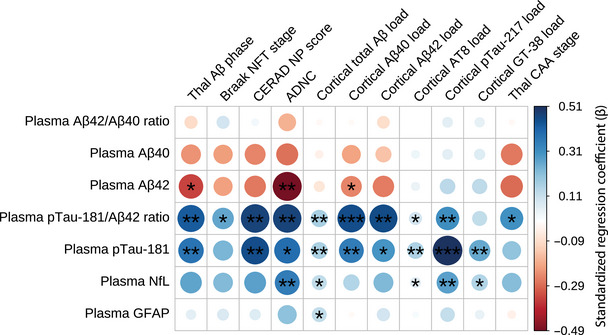
Associations between plasma biomarker concentrations and neuropathological substrates. Robust linear regression analyses between plasma biomarkers (rows: amyloid beta (Aβ)42/40 ratio; Aβ42; Aβ40; phosphorylated tau‐181 (pTau‐181)/Aβ42 ratio; pTau‐181; neurofilament light (NfL); glial fibrillary acidic protein (GFAP)) and neuropathological substrates (columns: Thal phase, Braak neurofibrillary tangle (NFT) stage, Consortium to Establish a Registry for Alzheimer's Disease Neuritic Plaque score (CERAD NP score), Alzheimer's Disease Neuropathologic Change (ADNC), cortical total Aβ load, cortical Aβ40 load, cortical Aβ42 load, cortical phosphorylated tau 202/205 load (cortical AT8 load), cortical phosphorylated tau‐217 load (Cortical pTau‐217 load), cortical GT‐38 load, and Thal cerebral amyloid angiopathy stage (Thal CAA stage)). The color scale on the right represents correlation coefficients, ranging from −0.49 (negative correlation, red) to 0.51 (positive correlation, blue), with larger circles indicating a stronger magnitude of correlation. All variables were standardized (*z*‐scored) to ensure comparability between the regression coefficients. Robust linear regression models were employed to calculate models. To account for potential heteroscedasticity in the residuals, robust standard errors were calculated using the heteroscedasticity correction (HC2) estimator. Models were corrected for age at blood collection, sex, the time difference between blood and brain donation, TDP‐43 stage, Braak LB stage, atherosclerosis, cerebral atrophy, hippocampal sclerosis, and the presence of infarcts. Asterisks denote statistically significant associations at a predefined significance level. *p* values (*≤0.10, **≤0.05, ***≤0.01) were adjusted for false discovery rate (FDR) using the Benjamini–Hochberg method and scaled by the effective number of independent tests (M_eff_) estimated via the Li & Ji method. *n* = 44 to 60. Numerical regression coefficients and *p* values can be found in Table S6.

**FIGURE 4:**
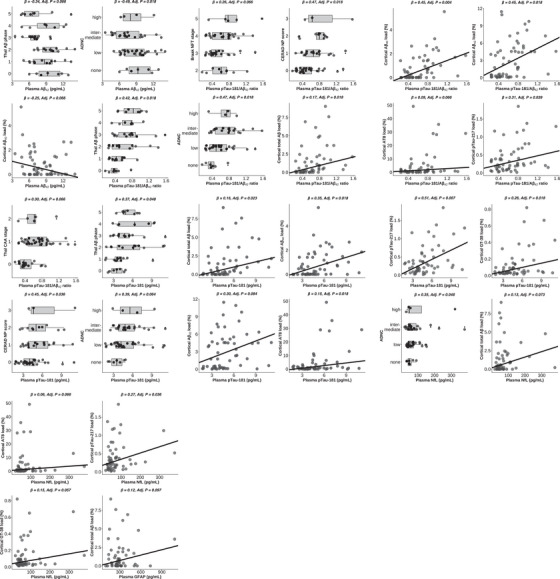
Scatterplots of associations between plasma biomarkers and neuropathological substrates. Each panel presents a robust linear regression fit (solid line) along with the standardized regression coefficient (*β*) and *p* value for predictor variable (plasma biomarker). *n* = 44 to 60.

*Post mortem* brain donation (*n* = 60) occurred a median of 16.1 months (IQR 3.9 to 28.4) [0.1 to 61.4] after blood collection (Table 2). Associations between plasma biomarkers and *post mortem* Aβ and tau neuropathology were adjusted for co‐pathologies of TDP‐43 stage, Braak LB stage, atherosclerosis, cortical atrophy, hippocampal sclerosis, and infarcts. As detailed in Table S5, these co‐pathologies did not account for significant variance in plasma biomarker concentrations. Lower Aβ40 and Aβ42 concentrations were consistently associated with higher Aβ neuropathology (Figure 3 and Table S6). Specifically, lower plasma Aβ42 was significantly associated with a higher Thal Aβ phase (*β =* −0.34, *Adj.P* = 0.066), higher ADNC score (*β =* −0.49, *Adj.P* = 0.018), and a greater quantitative cortical Aβ40 load (*β =* −0.25, *Adj.P* = 0.066; Figure 4). A higher plasma pTau‐181/Aβ42 ratio was associated with a higher Thal Aβ phase (*β =* 0.42, *Adj.P*  = 0.018), Braak NFT stage (*β =* 0.26, *Adj.P*  = 0.066), CERAD NP score (*β =* 0.47, *Adj.P* = 0.018), ADNC score (*β =* 0.47, *Adj.P* = 0.018), and higher cortical loads of total Aβ (*β =* 0.17, *Adj.P* = 0.018), Aβ40 (*β =* 0.45, *Adj.P* = 0.004), Aβ42 (*β =* 0.45, *Adj.P* = 0.018), AT8 (*β =* 0.09, *Adj.P* = 0.066) and pTau‐217 (*β =* 0.31, *Adj.P *= 0.039). Higher plasma pTau‐181 was associated with a higher Thal Aβ phase (*β =* 0.37, *Adj.P*  = 0.048), CERAD NP score (*β =* 0.45, *Adj.P* = 0.036), ADNC score (*β =* 0.39, *Adj.P* = 0.064), and higher cortical loads of total Aβ (*β =* 0.16, *Adj.P* = 0.023), Aβ40 (*β =* 0.35, *Adj.P* = 0.018), Aβ42 (*β =* 0.30, *Adj.P* = 0.084), AT8 (*β =* 0.15, *Adj.P* = 0.018), pTau‐217 (*β =* 0.51, *Adj.P* = 0.007), and GT‐38 (*β =* 0.26, *Adj.P *= 0.018). Higher plasma NfL associated with higher ADNC score (*β =* 0.35, *Adj.P* = 0.048), cortical loads of total Aβ (*β =* 0.13, *Adj.P* = 0.073), AT8 (*β =* 0.06, *Adj.P* = 0.066), pTau‐217 (*β =* 0.27, *Adj.P* = 0.036), and GT‐38 (*β =* 0.15, *Adj.P* = 0.057). Higher GFAP was associated with higher cortical loads of total Aβ (*β =* 0.12, *Adj.P* = 0.097). In contrast, the plasma Aβ42/40 ratio was not associated with Aβ and tau neuropathology.

## 4 DISCUSSION

This study provides unique insights into the complex interplay between AD‐associated plasma biomarkers, cognitive performance, and *post mortem* neuropathology in centenarians. Plasma biomarker concentrations of Aβ40, Aβ42, NfL, and GFAP are associated with both cognitive performance and *post mortem* neuropathology. In stark contrast, while plasma pTau‐181 and the pTau‐181/Aβ42 ratio were robustly associated with both Aβ and tau neuropathology, they showed no associations with cognitive performance in this cohort. We hypothesize that more pronounced tau accumulation may be required for plasma pTau‐181 concentrations to reach levels where an association with cognitive performance becomes observable. Lastly, our findings suggest that the value of the Aβ42/40 ratio as a reflection of cognitive decline may be attenuated at extreme age.

The robust link of plasma pTau‐181 with tau neuropathology recapitulates observations in younger AD patients and cognitively healthy individuals.^8^, ^9^, ^10^, ^40^, ^41^, ^42^ In our cohort, we observed the strongest associations for pTau‐181 with the tau pathology burden in the temporal cortex, as measured by three markers spanning the progression from pre‐tangles to ghost tangles. Indeed, increased plasma pTau‐181 concentrations are consistently observed in AD patients with advanced AD neuropathology, both cross‐sectionally and longitudinally,^43^, ^44^, ^45^, ^46^ while association with cognitive performance in younger individuals without cognitive decline remains inconsistent.^45^, ^47^, ^48^, ^49^, ^50^, ^51^ We speculate that for plasma pTau‐181 concentrations to associate with cognitive performance, a higher cortical p‐Tau abundance is required than was observed in this cohort.

Relative to pTau‐181, the pTau‐181/Aβ42 ratio demonstrated a stronger association with Aβ neuropathology in our cohort. This finding aligns with the amyloid cascade hypothesis, which states that Aβ accumulation instigates downstream tau pathology.^52^ This makes the pTau‐181/Aβ42 ratio a more potent marker for detecting Aβ neuropathology than either pTau‐181 or Aβ42 alone. Possibly, the sensitivity of the pTau‐181/Aβ42 ratio may also better capture the vascular deposition of amyloid as we observed a positive association between pTau‐181/Aβ42 ratio and Thal stage for CAA that we did not observe with pTau‐181 or Aβ42 separately. This aligns with Chong et al. (2021), who demonstrated that the pTau‐181/Aβ42 ratio was the strongest predictor of brain amyloid burden (Aβ‐PET) in a cohort with a high baseline of cerebrovascular disease (CeVD). Notably, they found no association with general CeVD markers like lacunes or white matter hyperintensities, underscoring its specificity for amyloid‐driven processes.^53^

Lower plasma Aβ40 and Aβ42 levels were associated with higher levels of Aβ neuropathology and with poorer performance across a range of cognitive tests, particularly in the domain of executive functioning. A previous study reported a positive association of plasma Aβ40, Aβ42, and the Aβ42/40 ratio with memory and of Aβ42 and the Aβ42/40 ratio with attention and global cognitive function,^43^ while other studies did not observe such associations.^54^, ^55^, ^56^, ^57^ Intriguingly, we recently observed a strong association between increased quantitative cortical Aβ load and poorer cognitive performance in the same cohort, especially within the executive functioning domain.^27^ Taken together, these findings suggest that plasma Aβ concentrations may indeed serve as a proxy for cortical Aβ deposition. However, caution is warranted as >90% of Aβ measured in plasma is estimated to be derived from peripheral sources,^58^ and AD patients typically show only a modest 10% to 15% decrease in total plasma Aβ compared to cognitively unimpaired individuals,^59^ making the precise pathophysiological link complex.

In our centenarian cohort, the plasma Aβ42/40 ratio did not correlate with either Aβ or tau neuropathology, unlike studies in younger elderly and cognitively impaired individuals that report weak but significant associations.^15^, ^40^, ^60^, ^61^, ^62^ A possible explanation for this may be that aging disproportionally elevates plasma Aβ40​ relative to Aβ42,^63^ distorting the Aβ42/40 ratio independently of Aβ42​ sequestration into Aβ plaques, and undermining its reliability as an indicator of AD pathology at extreme age.^64^ Alternatively, concentrations of plasma Aβ40 and Aβ42 ​may be sensitive enough to capture the initial changes of cognitive decline affecting this cohort,^65^ whereas the Aβ42/40 ratio may only become informative in more advanced neurodegenerative stages.

Of all biomarkers measured, elevated plasma GFAP was most strongly associated with poorer cognitive performance, specifically executive functioning, attention/processing speed, the last of which is particularly sensitive to aging.^66^, ^67^ This aligns with findings in younger cohorts.^48^, ^68^, ^69^ However, plasma GFAP showed little association with Aβ or tau neuropathology in our cohort, contrasting with reports in younger elderly and AD patients using tau‐PET imaging or *post mortem* data.^70^, ^71^, ^72^ We therefore propose that, in the oldest old, elevated plasma GFAP may reflect age‐related rather than AD‐related cognitive decline. As with the Aβ42/40 ratio, the value of GFAP as a marker for neuropathology may emerge with more advanced neurodegeneration. Such preliminary signals need replication in independent datasets, and mechanistic studies will be essential.

Elevated plasma NfL was associated with higher Aβ and tau neuropathology and showed weak but consistent associations with lower cognitive performance, which is consistent with findings in younger cognitively healthy elderly and in AD patients.^73^, ^74^, ^75^ While NfL is a general marker of axonal damage not specific to AD,^76^, ^77^ our findings suggest that associated neuronal injury occurs alongside Aβ and tau neuropathology, also in centenarians. The modest link to cognitive performance might indicate either that neuronal injury is insufficient to cause major cognitive impairment or that compensatory mechanisms preserve cognitive function in these resilient individuals.

To our knowledge, this is the first study integrating plasma biomarkers, comprehensive cognitive testing, and a broad range of Aβ and tau neuropathology substrates in centenarians spanning intact cognition to early cognitive impairment. While a cohort of 60 centenarians with matched plasma, cognitive, and *post mortem* neuropathology data is unprecedented, replication in larger oldest old cohorts is warranted. The 100‐plus Study's inclusion criteria – 100 years or older and self‐reported as cognitively healthy – limit generalizability, as our participants are likely resistant or resilient to certain dementia risk factors. Generalizability is further limited as our cohort includes mostly individuals of Northwestern European ancestry. Furthermore, the home‐visit setting of our study precludes control over pre‐analytical factors (e.g., diet, temperature, collection time, centrifugation delay). Also, we could not control for specific systemic conditions that can influence biomarker concentrations, such as chronic kidney disease, known to reduce NfL clearance.^78^ While no participants were on dialysis, we cannot exclude that non‐cerebral factors influenced biomarker concentrations. AD neuropathology and concurrent symptoms are known to accumulate over decades,^79^ such that any AD pathology in this group had likely already accumulated by the age of 100. However, we cannot exclude that any changes occurred during the median 16.1‐month time interval between the collection of plasma samples and *post mortem* assessment. Lastly, to avoid bias toward the best‐performing centenarians, we applied MICE to handle missing cognitive data from participants who did not complete the full test battery, instead of performing complete‐case analysis. This approach is widely recognized as an appropriate method for addressing missing neuropsychological data, particularly in AD research.^80^, ^81^ Despite these challenges, our unique study design offered a rare opportunity to observe the earliest stages of decline in exceptionally long‐lived individuals, which would require decades of follow‐up in younger cohorts.

Future work should expand the plasma panel to include biomarkers like plasma pTau‐231 and plasma pTau‐217,^9^, ^82^ as well as plasma biomarkers for age‐related comorbidities,^83^ including limbic‐predominant age‐related TDP‐43 encephalopathy (LATE)^84^ and vascular neuropathology (e.g., infarcts, atherosclerosis), along with determinants of amyloid subtypes (e.g., diffuse and cored plaques, CAA). While effect sizes were consistent, even in our relatively small cohort, a further exploration might prefer spectrometry‐based rather than immunoassay‐based plasma marker quantification.^60^ Analyses could also benefit from correlating plasma biomarkers with neuropathology in multiple brain regions, rather than solely the temporal cortex. We chose the temporal pole because it enabled simultaneous quantification of both Aβ and tau, as many centenarians exhibit exclusively cortical, and not hippocampal, amyloid deposition.

In conclusion, our findings in centenarians reveal differential sensitivities of AD‐associated plasma biomarkers for detecting changes in cognitive performance and AD‐associated neuropathology. On the one hand, plasma GFAP is strongly associated with cognitive function but not with AD‐specific neuropathology, suggesting it may reflect more general age‐related processes. On the other hand, even though the potent pTau‐181/Aβ42 ratio and pTau‐181 are robust indicators of underlying Aβ and tau pathology, they showed no associations with cognitive performance. While this may be a reflection of the relatively low tau levels present in this cohort, precluding any robust association with cognitive performance, our findings provide unique insights into the complex relationships between plasma biomarkers, neuropathology, and the potential to reflect cognitive performance.

## CONFLICT OF INTEREST STATEMENT

The authors declare that they have no competing interests. J.J.M.H. is currently employed by F. Hoffmann‐La Roche. Author disclosures are available in the Supporting Information.

## CONSENT STATEMENT

All human subjects provided written informed consent prior to participation in the study.

## Supporting information



Supporting information

Supporting information
