# Supplementary material for: AD‐related plasma biomarkers in centenarians: links to cognition and neuropathology
Source: Alzheimers Dement. 2025 Dec 19;21(12):e70969. doi: 10.1002/alz.70969 (PMC12715708; doi:10.1002/alz.70969)
Supplement: Supplementary file 1 — Supporting information [file ALZ-21-e70969-s001.docx]

***SUPPLEMENTARY MATERIAL*

AD-related plasma biomarkers in centenarians: links to cognition and neuropathology**

**TABLE OF CONTENTS
1 SUPPLEMENTARY METHODS**

**1.1 Study design**

- - 1. *Procedure and participants*

**1.2 Blood-based biomarker evaluation**

- - 1. *Blood collection*
    2. *Simoa plasma biomarker measurements*

**1.3 Cognitive test data**

- - 1. *Neuropsychological test battery*
    2. *Missing cognitive test score imputation*

**1.4 Neuropathological evaluation**

- - 1. *Immunohistochemistry and neuropathological assessment*
    2. *Thal Aβ phase*
    3. *Braak NFT stage*
    4. *CERAD-NP score*
    5. *CAA stage*
    6. *TDP-43 stage*
    7. *Quantitative neuropathology*

**2 SUPPLEMENTARY FIGURES**

*2.1 Supplementary Figure 1 Venn diagram of centenarians across different assessments*

*2.2* *Supplementary Figure 2 Distribution of neuropathological substrates*

*2.3 Supplementary Figure 3 Quantitative Aβ and tau pathology*

*2.4 Supplementary Figure 4 Raw distributions of cognitive test scores*

**3 SUPPLEMENTARY TABLES**

*3.1 Supplementary Table 1 Robust linear regression associations between plasma biomarkers and cognitive performance with Imputation-Derived RIV and FMI Statistics*

*3.2 Supplementary Table 2 Cognitive test imputation*

*3.3 Supplementary Table 3 Antibody and antigen retrieval details for immunohistochemistry*

*3.4 Supplementary Table 4. Robust linear regression associations between plasma biomarkers and cognitive performance*

*3.5 Supplementary Table 5 Robust linear regression associations between plasma biomarkers and measures of neuropathological covariates*

*3.6 Supplementary Table 6 Robust linear regression associations between plasma biomarkers and measures of neuropathological substrates*

**REFERENCES**

**1 SUPPLEMENTARY METHODS**

1.1 Study design

1.1.1 Procedure and participants

The 100-plus Study is an ongoing longitudinal prospective cohort study comprising of centenarians who self-report to be cognitively healthy at baseline, as confirmed by proxy. Detailed descriptions of the cohort’s collection procedures and characteristics have been published previously.^1^ All centenarians were born between 1909 and 1923 and had no clinical diagnosis of dementia or signs of neurological disorders at the time of inclusion. Baseline home visits involved collecting comprehensive lifetime histories, including educational backgrounds, assessing cognitive performance and physical functioning, and collecting blood samples. Postmortem brain donation was optional. Home visits and blood collection typically occurred on the same day. Among the centenarians, cognitive assessments were available for 472 centenarians, of whom 111 had donated their brain (**Figure S1**).

1.2 Blood-based biomarker evaluation

1.2.1 Blood collection

Samples were allowed to clot at room temperature during transit to the lab, where they were centrifuged at 1800 x g for 10 minutes, plasma was aliquoted into 0.5 mL aliquots in 1.5-2.0 mL polypropylene tubes (Sarstedt, Germany), and stored at -80°C in the Amsterdam UMC Biobank. Sample preparation involved thawing at room temperature followed by centrifugation at 10,000 x g and 4°C for five minutes, with the supernatant subsequently used for assays.

1.2.2 Simoa plasma biomarker measurements

All plasma samples were labeled with a random code and randomized across assay batches. All measured biomarker concentrations were above the lower limit of quantification (LLOQ), ensuring reliable quantification. For pTau-181, intra-assay and inter-assay variability were maintained within acceptable limits, with coefficient of variation (CV) percentages of 6.1% and 9.2% for low quality control (QC), and 11.4% for high QC, respectively. Quality control procedures included the analyses of two control samples per biomarker on each plate, both at the start and end, achieving a CV% below 20%, indicating consistent assay performance. Given GFAP's sensitivity to multiple freeze-thaw cycles, data from three participants with 3 freeze-thaw cycles were excluded to preserve integrity.

1.3 Cognitive test data

1.3.1 Neuropsychological test battery

Global cognitive functioning was assessed using the Mini-Mental State Examination (MMSE)^2,3^ (scores ranging from 0-30, higher is better). Memory was evaluated using the reproduction and recall components of the Consortium to Establish a Registry for Alzheimer’s Disease (CERAD) 10 word test^4^ and the first two trials of the Visual Association Test (VAT).^5^ Executive functions were evaluated with the Wechsler Adult Intelligence Scale third edition (WAIS-III) Digit Span Backward subtest,^6^ the Behavioural Assessment of the Dysexecutive Syndrome (BADS) Key Search test,^7^ the Trail making Test B (TMT-B)^8^ and the Clock drawing test (CDT), scored according to the Shulman scoring system.^9,10^ Attention and processing speed were measured using the Trail making Test A (TMT-A)^8^ and the WAIS-III Digit Span Forward subtest.^6^ Verbal fluency was assessed with the naming section of the VAT, the Dutch version of the Controlled Oral Word Association Test (COWAT) for animal naming, and the letter fluency test (naming words starting with the letters D, A, and T; **Table S2**).^11-13^

1.3.2 Missing cognitive test score imputation

For the MICE procedure, we utilized data from all 472 centenarians included in the study at the time of analysis. This included age at cognitive assessment, sex, all available neuropsychological test scores, Geriatric Depression Scale (GDS) scores,^14^ Barthel Index (BI) scores for activities of daily living (ADL) functioning,^15^ vision and hearing abilities, educational attainment, Informant Questionnaire on Cognitive Decline in the Elderly (IQCODE) scores,^16^ test time limit exceedances and qualitative evaluations of cognitive status, understanding, and motivation to complete the tests. Missing MMSE items were individually imputed, accounting for sensory function variability among centenarians. Records ≥6 missing responses from the MMSE were excluded from the analyses. To maximize statistical power of our test-sample, maintain a consistent sample size across all tests, and to minimize any bias towards the best-performing centenarians we imputed missing neuropsychological test scores.

1.4 Neuropathological evaluation

1.4.1 Immunohistochemistry and neuropathological assessment

Sections were deparaffinized and rehydrated in a series of xylene and ethanol followed by antigen retrieval**.** Thereafter, sections were rinsed in phosphate-buffered saline (PBS) three times. The sections were incubated with the primary antibody diluted in Antibody Diluent (Invitrogen, # 10414753) (**Table S3**) overnight at room temperature. Sections were rinsed thrice in PBS and incubated with EnVision (anti-mouse/rabbit HRP, DAKO, Glostrup, Denmark, #K5007) for 30 minutes at room temperature and washed thrice. This was followed by treatment with 3,3'-diaminobenzidine (DAB) for 5 minutes. Nuclei were counterstained with hematoxylin, followed by dehydration in a series of alcohol and xylene.

1.4.2 Thal Amyloid-βeta (Aβ) phase

The spatiotemporal distribution of Aβ pathology was assessed using the Thal phase system, which ranges from 1 to 5.^17^ The presence of Aβ plaques was evaluated in specific brain regions: middle frontal gyrus, middle temporal cortex, temporal pole cortex, inferior parietal lobule cortex, occipital pole cortex. If no Aβ plaques of any kind nor cerebral amyloid angiopathy (CAA) were observed, Thal phase 0 was assigned. If any number of Aβ plaques of any kind were observed in any of these regions, but none of the following, a Thal phase of 1 was assigned; CA1 region of the hippocampus and entorhinal cortex (Thal phase 2); dentate gyrus, presubiculum, amygdala, nucleus caudatus, putamen and nucleus accumbens (Thal phase 3); CA4 region of the hippocampus, substantia nigra and inferior olivary nucleus (Thal phase 4); cerebellum (Thal phase 5).

1.4.3 Braak neurofibrillary tangles (NFT) stage

The distribution of neurofibrillary tangles (NFTs) was evaluated according to the Braak stages (0-VI).^18^ The presence of NFTs stained by Gallyas silver and AT8 were evaluated in the following brain regions: stage I-II: transentorhinal and entorhinal cortex; III-IV: hippocampus; V-VI: middle frontal cortex, middle temporal cortex, temporal pole cortex, inferior parietal lobule, occipital pole cortex.

1.4.4 CERAD- Neuritic plaque (NP) score

The semi-quantitative frequency of neuritic plaques (NPs) was evaluated according to the Consortium to Establish a Registry for Alzheimer’s Disease (CERAD) guidelines^19^. The middle frontal gyrus, inferior parietal lobe and middle temporal gyrus were stained with Gallyas Silver. Neuritc plaques were scores as absent (0), sparse (1), moderate (2) or frequent (3).

1.4.5 Cerebral amyloid angiopathy (CAA) stage

The distribution of CAA was assessed according to the Thal stages (0-3)^20^. Stage 1 was assigned when CAA was restricted to the leptomeninges and cortical vessels of the neocortex. Stage 2 was assigned when CAA was additionally present in the vessel of the allocortex, cerebellum or midbrain. Stage 3 was assigned when CAA was additionally observed in the lower brainstem, basal ganglia or thalamus.

1.4.6 TAR-DNA binding protein of 43 (TDP-43) stage

The distribution of TDP-43 pathology was evaluated in the amygdala (stage 1), hippocampus (stage 2), and the neocortex of the middle frontal gyrus (stage 3).^21^ The distributions of all neuropathological substrates are detailed in **Figure S2**.

1.4.7 Quantitative neuropathology

The temporal cortex was selected due to its early involvement in Aβ pathology (Thal phase 1) and significant tau pathology observed in the centenarian cohort. In brief, 6 µm thick formalin-fixed paraffin-embedded (FFPE) sections were stained with several Aβ and tau antibodies and scanned at 20x magnification using an Olympus VS200 slide scanner with VS200 ASW software (version 3.3), (**Figure S3**). Digital scans were analyzed using the QuPath open-source software (version 0.3.2).^22^ Grey matter regions were manually annotated as regions of interest (ROIs). An artificial neural network was trained on a set of 10 images representing the full spectrum of immunoreactivity in terms of frequency and intensity. Pathology load for each image was quantified as the percentage of grey matter positive for the target protein.

2 SUPPLEMENTARY FIGURES

|  |
| --- |
| **Supplementary Figure 1. Venn diagram of centenarian participants across different assessments.** This Venn diagram illustrates the distribution and overlaps of centenarian participants across three key assessments: cognitive performance, plasma biomarkers, and *postmortem* brain donations. Cognitive performance was assessed in 472 participants, plasma biomarker levels were measured in 255 participants, and complete neuropathological characterization was available for 111 participants. The intersections of the circles indicate the number of participants who underwent multiple assessments. |

| 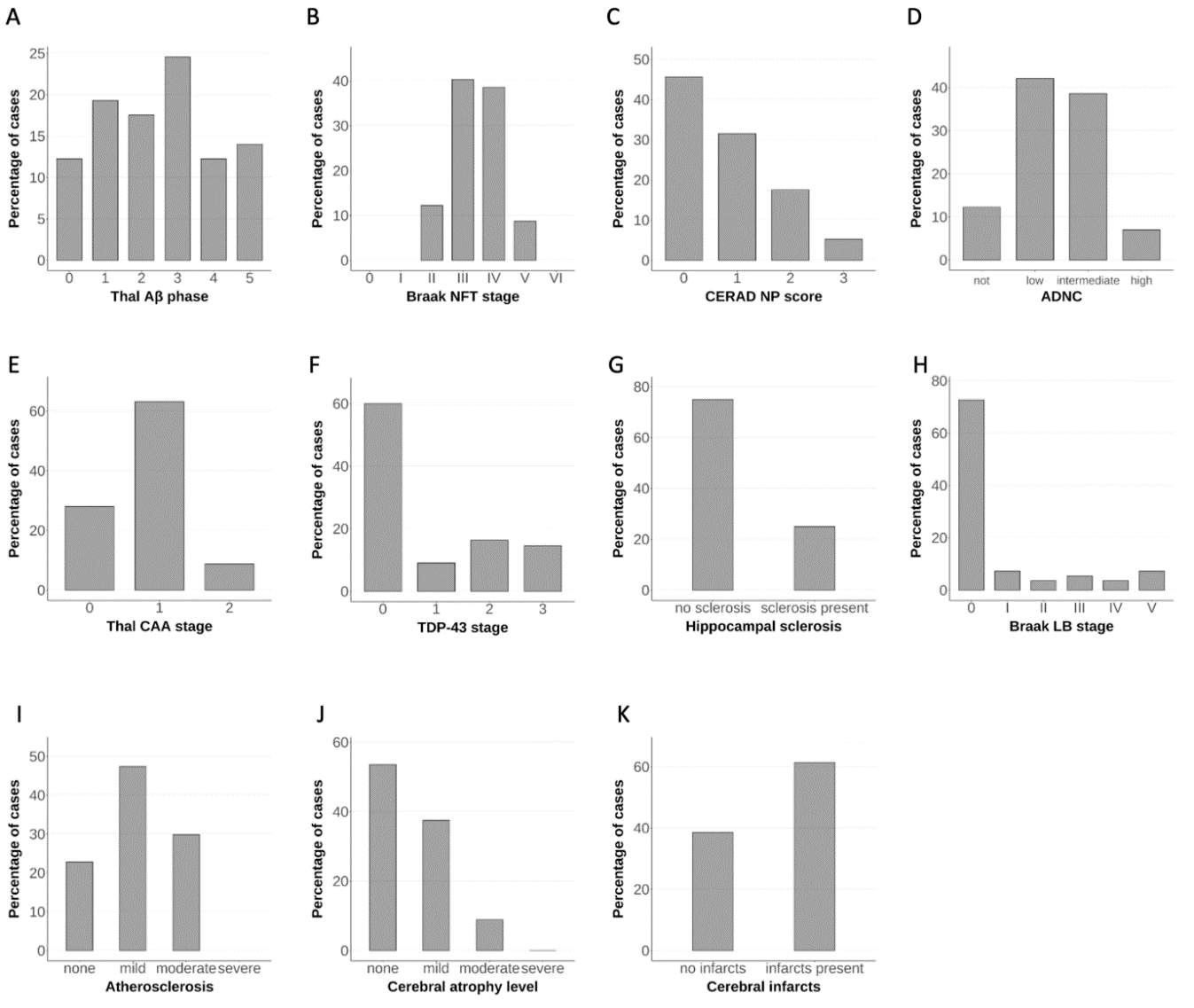 |
| --- |
| **Supplementary Figure 2**. **Distribution of neuropathological substrates.** Bar plots representing the percentage distribution of various neuropathological features. (A) Thal Amyloid-β (Aβ) phase; (B) Braak Neurofibrillary Tangle (NFT) stage; (C) CERAD Neuritic Plaque (NP) score; (D) Alzheimer’s Disease Neuropathologic Change (ADNC) score; (E) Thal Cerebral Amyloid Angiopathy (CAA) stage; (F) TAR DNA-binding Protein 43 (TDP-43) stage; (G) Presence of hippocampal sclerosis; (H) Braak Lewy Body (LB) stage; (I) Atherosclerosis severity; (J) Cerebral atrophy level; (K) Presence of cerebral infarcts. |

| 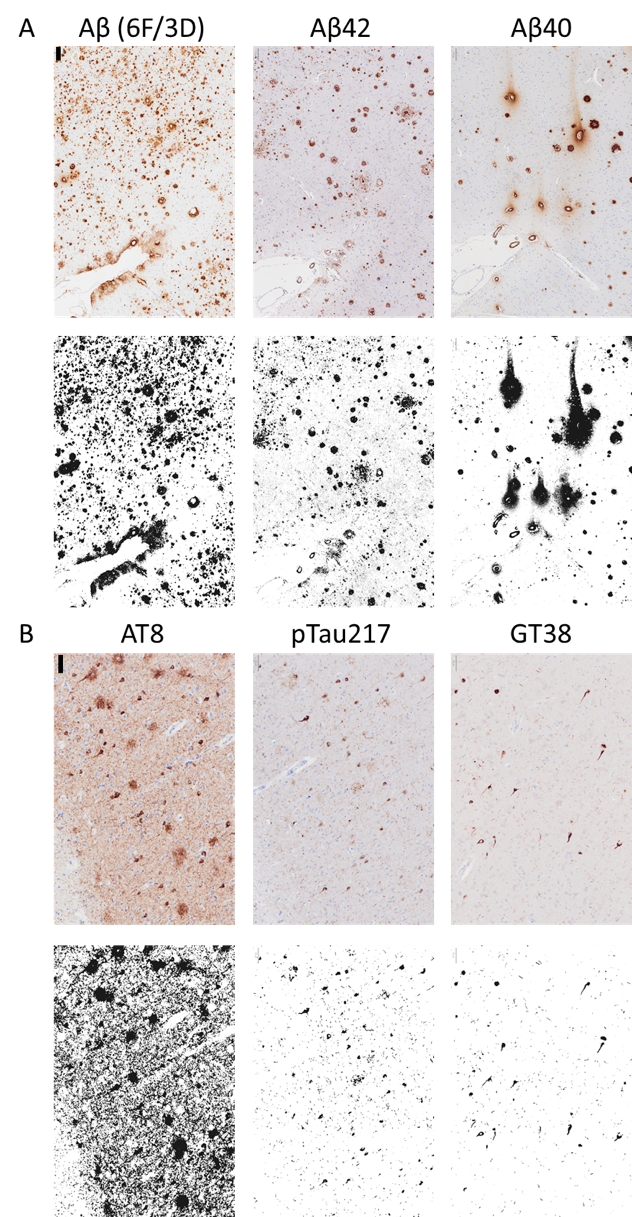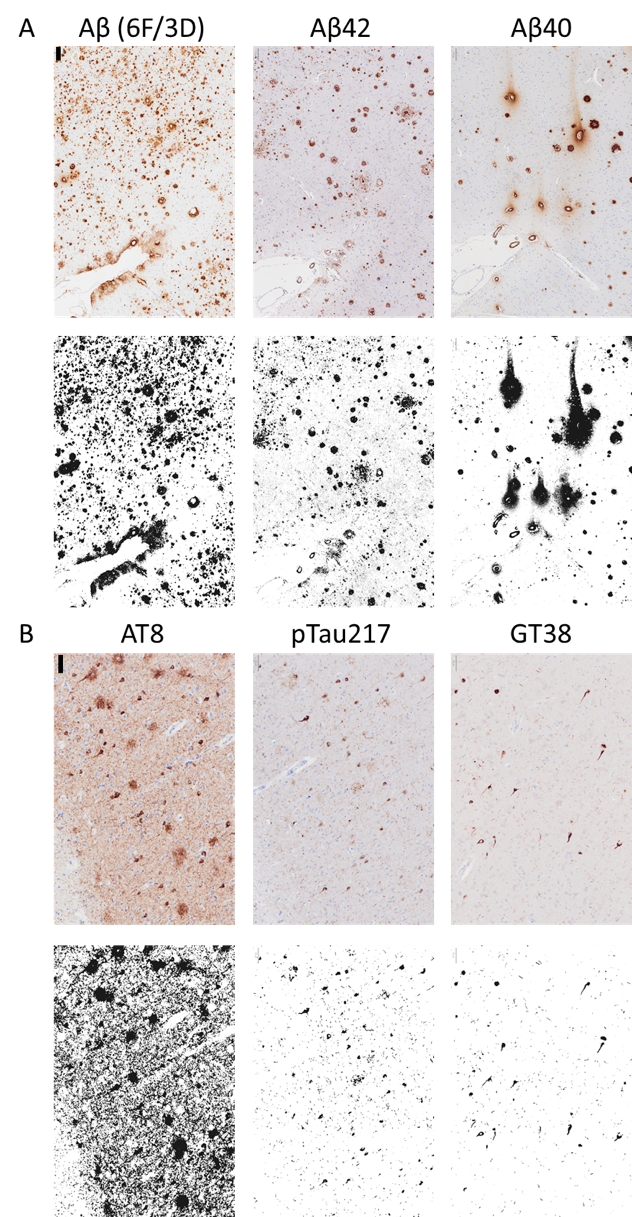 |
| --- |
| Supplementary Figure 3. Quantitative Aβ and tau pathology. Abbreviations: Aβ - Amyloid-beta; AT8 - Phosphorylated Tau at positions 202/205 (pre-tangles); GT-38 - Mature to ghost tangle marker ; pTau-217 - Phosphorylated Tau at position 217 (intermediate to mature tangles). (A) Images showing immunohistochemical staining for total Aβ (6F/3D), Aβ40, and Aβ42 in the temporal pole from one centenarian donor with visualization of detection of immunopositivity by a pixel classifier. Scale bar 100 µm. (B) Images showing immunohistochemical staining for different stages of tau pathology in the temporal pole. The markers visualize pre-tangles (AT8), intermediate to mature tangles (pTau-217), and mature to ghost tangles (GT-38). The lower panel displays the corresponding immunopositivity. Scale bar 50 µm. |

| **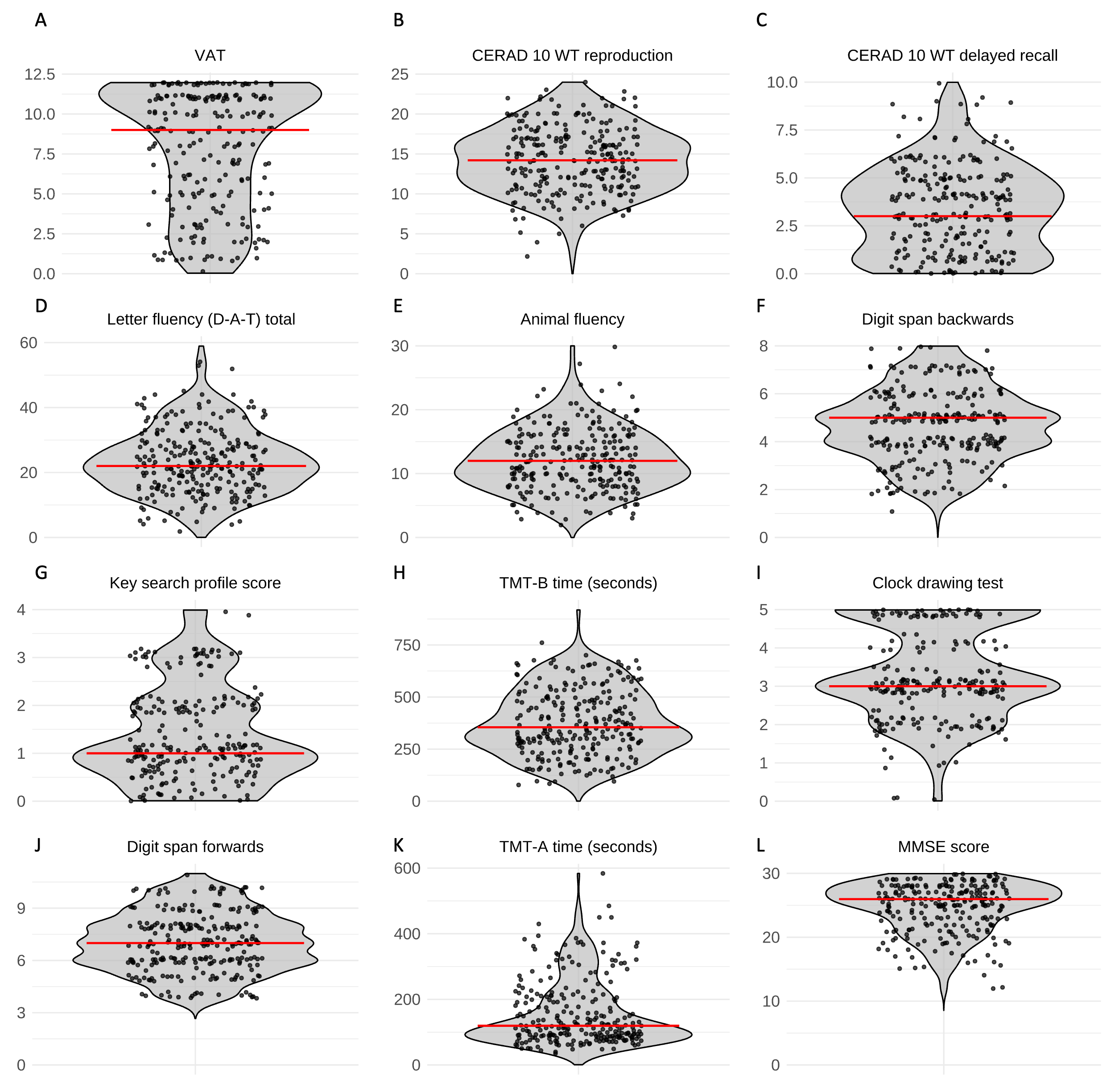** |
| --- |
| **Supplementary Figure 4**. **Raw distributions of cognitive test scores.** Violin plots representing the distribution of various cognitive tests. (A) Visual Association Test (VAT); (B) Consortium to Establish a Registry for Alzheimer’s Disease (CERAD) 10 word test reproduction; (C) CERAD 10 word test delayed recall; (D) Controlled Oral Word Association Test (COWAT) Letter fluency (letters D-A-T); (E) COWAT animal fluency (1 minute); (F) WAIS-III Digit Span Backwards subtest; (G) Behavioural Assessment of the Dysexecutive Syndrome (BADS) Key Search test; (H) Trail making Test B (TMT-B); (I) Clock drawing test (CDT); (J) WAIS-III Digit Span Forward subtest; (K) TMT-A test; (L) Mini-Mental State Examination (MMSE). The red line represents the median value. N=243-255. |

3 SUPPLEMENTARY TABLES

| **Supplementary Table 1. Robust linear regression associations between plasma biomarkers and cognitive performance with imputation-derived RIV and FMI** | | | | | | | | | | | | | |
| --- | --- | --- | --- | --- | --- | --- | --- | --- | --- | --- | --- | --- | --- |
|  |  | **Visual Association Test (VAT)** | **CERAD 10 WT reproduction** | **CERAD 10 WT delayed recall** | **Letter fluency (D-A-T)** | **Animal fluency** | **Digit span backwards** | **Key search test** | **TMT-B time (reversed)** | **Clock drawing test** | **Digit span forward** | **TMT-A time**  **(reversed)** | **MMSE score** |
| **Aβ_42/40_ ratio** | ***RIV*** | 0.070 | 0.210 | 0.106 | 0.211 | 0.043 | 0.124 | 0.210 | 0.256 | 0.100 | 0.150 | 0.207 | 0.025 |
|  | ***FMI*** | 0.066 | 0.176 | 0.097 | 0.177 | 0.042 | 0.112 | 0.176 | 0.207 | 0.092 | 0.132 | 0.174 | 0.024 |
| **Aβ_40_** | ***RIV*** | 0.070 | 0.203 | 0.146 | 0.096 | 0.055 | 0.113 | 0.506 | 0.199 | 0.124 | 0.150 | 0.115 | 0.032 |
|  | ***FMI*** | 0.066 | 0.171 | 0.129 | 0.089 | 0.053 | 0.103 | 0.343 | 0.168 | 0.112 | 0.132 | 0.104 | 0.031 |
| **Aβ_42_** | ***RIV*** | 0.057 | 0.243 | 0.112 | 0.161 | 0.053 | 0.125 | 0.363 | 0.268 | 0.055 | 0.172 | 0.076 | 0.025 |
|  | ***FMI*** | 0.054 | 0.198 | 0.102 | 0.140 | 0.051 | 0.112 | 0.271 | 0.215 | 0.052 | 0.149 | 0.071 | 0.024 |
| **pTau-181/Aβ_42_** | ***RIV*** | 0.031 | 0.186 | 0.166 | 0.176 | 0.076 | 0.099 | 0.186 | 0.324 | 0.146 | 0.240 | 0.121 | 0.021 |
|  | ***FMI*** | 0.031 | 0.159 | 0.144 | 0.152 | 0.072 | 0.091 | 0.159 | 0.249 | 0.129 | 0.196 | 0.109 | 0.020 |
| **pTau-181** | ***RIV*** | 0.027 | 0.280 | 0.137 | 0.198 | 0.140 | 0.196 | 0.447 | 0.417 | 0.194 | 0.222 | 0.100 | 0.033 |
|  | ***FMI*** | 0.026 | 0.222 | 0.122 | 0.167 | 0.124 | 0.166 | 0.315 | 0.300 | 0.165 | 0.184 | 0.092 | 0.032 |
| **NfL** | ***RIV*** | 0.078 | 0.502 | 0.438 | 0.213 | 0.211 | 0.293 | 0.461 | 0.641 | 0.272 | 0.314 | 0.641 | 0.029 |
|  | ***FMI*** | 0.073 | 0.340 | 0.310 | 0.178 | 0.177 | 0.230 | 0.321 | 0.398 | 0.217 | 0.243 | 0.398 | 0.029 |
| **GFAP** | ***RIV*** | 0.098 | 0.653 | 0.229 | 0.146 | 0.101 | 0.153 | 0.316 | 0.298 | 0.137 | 0.233 | 0.215 | 0.031 |
|  | ***FMI*** | 0.090 | 0.403 | 0.189 | 0.129 | 0.092 | 0.134 | 0.244 | 0.233 | 0.122 | 0.192 | 0.179 | 0.030 |
| Abbreviations: Aβ - Amyloid Beta; CERAD - Consortium to Establish a Registry for Alzheimer's Disease; CERAD 10 WT - Consortium to Establish a Registry for Alzheimer's Disease 10-Word Test; FMI - Fraction of Missing Information; GFAP - Glial Fibrillary Acidic Protein; MMSE - Mini-Mental State Examination; NfL - Neurofilament Light Chain; pTau-181 - Phosphorylated Tau at position 181; RIV - Relative Increase in Variance; TMT - Trail Making Test; VAT - Visual Association Test. Plasma biomarkers and cognitive tests: N=243-255. | | | | | | | | | | | | | |

| **Supplementary Table 2. Cognitive tests imputation** | | | | | | |
| --- | --- | --- | --- | --- | --- | --- |
| **Cognitive domain** | **Test** | **Subtest/scoring** | **Scoring range** | **Minimum scores obtained** | **Maximum scores obtained** | **Percentage of scores imputed** |
| **Memory (n=255)** | VAT | Trials 1+2 | 0–12 | 0 | 12 | 13 |
|  | CERAD 10 WT | Immediate recall | 0–30 | 2 | 24 | 20^a^ |
|  |  | Delayed recall | 0–10 | 0 | 10 | 21^a^ |
|  | | | | | | |
| **Fluency (n=255)** | Controlled Oral Word Association Test | Letter fluency  D-A-T  3 minutes total | not applicable | 2 | 59 | 19 |
|  |  | Animal fluency  1 minute | not applicable | 2 | 30 | 15 |
|  | | | | | | |
| **Executive functioning (n=255)** | WAIS III | Digit span backwards | 0–16 | 1 | 8 | 20 |
|  | BADS | Key search profile score | 0–4 | 0 | 4 | 38 |
|  | Trail Making Test B | Time in seconds | not applicable | 78 | 920^b^ | 46 |
|  | Clock Drawing Test | Shulman scoring | 0–5 | 0 | 5 | 19 |
|  | | | | | | |
| **Attention/processing speed (n=255)** | WAIS III | Digit span forward | 0–16 | 4 | 11 | 18 |
|  | Trail Making Test A | Time in seconds | not applicable | 35 | 584^b^ | 33 |
|  | | | | | | |
| **Global cognitive functioning (n=249)** | MMSE |  | 0–30 | 12 | 30 | 3.5 |
| Abbreviations: Animal Fluency, Behavioural Assessment of the Dysexecutive Syndrome (BADS) Key Search Test, CERAD 10-word list immediate reproduction (CERAD 10-word list), Clock Drawing Test, Letter Fluency (D-A-T), Mini-Mental State Examination (MMSE), Trail Making Test part A (TMT-A) and part B (TMT-B) times in seconds, Visual Association Test (VAT), and WAIS III digit span backward and forward. The minimum and maximum values represent the range of scores obtained within this cohort. ^a^ The Consortium to Establish a Registry for Alzheimer's Disease (CERAD) 10-word list was introduced into the neuropsychological battery at a later stage of the study. ^b^ For the Trail Making Test A and B, which are timed, lower scores indicate superior performance. | | | | | | |

| **Supplementary Table 3. Antibody and antigen retrieval details for immunohistochemistry** | | | | |
| --- | --- | --- | --- | --- |
| **Antibody** | **Catalogue number** | **Supplier** | **Dilution** | **Antigen retrieval** |
| Aβ(8-17) | M0872 (clone 6F/3D) | DAKO | 1:1000 | Citrate buffer + formic acid 80% |
| Aβ_40_ | MABN11 (clone G2-10) | Millipore | 1:1000 | Formic acid |
| Aβ_42_ | MABN12 (clone G2-11) | Millipore | 1:5000 | Formic acid |
| AT8 | MN1020 | Invitrogen | 1:800 | Citrate buffer |
| pTau-217 | 44-744 | Invitrogen | 1:2000 | Citrate buffer |
| GT-38 | Ab246808 | Abcam | 1:500 | Citrate buffer |
| Abbreviations: Aβ - Amyloid-beta; AT8 - Phosphorylated Tau at Ser202/Thr205; pTau-217 - Phosphorylated Tau at Threonine 217. Antigen retrieval was performed by using a pressure cooker to boil the tissue in citrate buffer (pH 6.0) for 20 minutes at 121°C, followed by rinsing with PBS, or by rinsing the tissue twice with distilled water and incubating with 80% formic acid for 5 minutes, followed by rinsing with PBS. Both methods were used for 6F/3D. | | | | |

| **Supplementary Table 4. Robust linear regression associations between plasma biomarkers and cognitive performance** | | | | | | | | | | | | | |
| --- | --- | --- | --- | --- | --- | --- | --- | --- | --- | --- | --- | --- | --- |
|  |  | **Visual Association Test (VAT)** | **CERAD 10 WT reproduction** | **CERAD 10 WT delayed recall** | **Letter fluency (D-A-T)** | **Animal fluency** | **Digit span backwards** | **Key search test** | **TMT-B time (reversed)** | **Clock drawing test** | **Digit span forward** | **TMT-A time**  **(reversed)** | **MMSE score** |
| **Aβ_42/40_ ratio** | ***β*** | 0.03 | 0.07 | -0.02 | 0.04 | 0.03 | 0.08 | -0.06 | 0.09 | 0.10 | 0.08 | 0.07 | 0.01 |
|  | ***Adj. P-value*** | 0.402 | 0.316 | 0.411 | 0.366 | 0.402 | 0.301 | 0.325 | 0.301 | 0.301 | 0.301 | 0.301 | 0.452 |
| **Aβ_40_** | ***β*** | 0.05 | 0.08 | 0.07 | 0.07 | 0.08 | 0.07 | 0.21 | 0.04 | 0.07 | 0.04 | 0.03 | 0.05 |
|  | ***Adj. P-value*** | 0.366 | 0.301 | 0.316 | 0.315 | 0.301 | 0.316 | **0.008** | 0.380 | 0.316 | 0.380 | 0.380 | 0.366 |
| **Aβ_42_** | ***β*** | 0.06 | 0.12 | 0.05 | 0.08 | 0.11 | 0.14 | 0.17 | 0.10 | 0.14 | 0.08 | 0.07 | 0.06 |
|  | ***Adj. P-value*** | 0.366 | 0.301 | 0.366 | 0.301 | 0.301 | 0.172 | **0.053** | 0.301 | 0.172 | 0.301 | 0.301 | 0.316 |
| **pTau-181/Aβ_42_** | ***β*** | 0.07 | -0.05 | -0.01 | 0.00 | 0.02 | -0.08 | -0.03 | 0.01 | -0.05 | -0.04 | 0.04 | 0.01 |
|  | ***Adj. P-value*** | 0.316 | 0.366 | 0.452 | 0.452 | 0.411 | 0.301 | 0.398 | 0.452 | 0.366 | 0.366 | 0.366 | 0.446 |
| **pTau-181** | ***β*** | 0.10 | 0.04 | 0.00 | 0.04 | 0.05 | 0.01 | 0.03 | 0.04 | 0.02 | 0.01 | 0.07 | 0.04 |
|  | ***Adj. P-value*** | 0.301 | 0.380 | 0.452 | 0.366 | 0.366 | 0.452 | 0.398 | 0.380 | 0.441 | 0.452 | 0.301 | 0.366 |
| **NfL** | ***β*** | -0.07 | -0.05 | -0.04 | -0.06 | -0.03 | -0.02 | 0.01 | -0.11 | -0.04 | -0.01 | -0.12 | -0.10 |
|  | ***Adj. P-value*** | 0.325 | 0.366 | 0.366 | 0.325 | 0.393 | 0.424 | 0.446 | 0.301 | 0.366 | 0.446 | 0.172 | 0.301 |
| **GFAP** | ***β*** | -0.09 | -0.10 | -0.14 | -0.11 | -0.08 | 0.01 | 0.01 | -0.19 | 0.04 | -0.01 | -0.18 | -0.10 |
|  | ***Adj. P-value*** | 0.301 | 0.301 | 0.172 | 0.301 | 0.301 | 0.446 | 0.452 | **0.053** | 0.380 | 0.452 | **0.024** | 0.301 |
| Abbreviations: Aβ - Amyloid-beta; CERAD - Consortium to Establish a Registry for Alzheimer's Disease; CERAD 10 WT - Consortium to Establish a Registry for Alzheimer's Disease 10-Word Test; GFAP - Glial Fibrillary Acidic Protein; MMSE - Mini-Mental State Examination; NfL - Neurofilament Light Chain; pTau-181 - Phosphorylated Tau at position 181; TMT - Trail Making Test; VAT - Visual Association Test. Plasma biomarkers and cognitive tests: N=243-255. *P*-values were adjusted for false discovery rate (FDR) using the Benjamini & Hochberg method and scaled by the effective number of independent tests (Mₑff) estimated via the Li & Ji method; significance was set at Adj. *P* < 0.10. | | | | | | | | | | | | | |

| **Supplementary Table 5. Robust linear regression associations between plasma biomarkers and measures of neuropathological covariates** | | | | | | | |
| --- | --- | --- | --- | --- | --- | --- | --- |
|  |  | **TDP-43 stage** | **Hippocampal sclerosis** | **Braak LB stage** | **Atherosclerosis** | **Cerebral atrophy** | **Cerebral infarcts** |
| **Aβ_42/40_ ratio** | ***β*** | -0.11 | 0.00 | 0.00 | -0.06 | 0.00 | -0.10 |
|  | ***Adj. P-value*** | 0.844 | 0.844 | 0.844 | 0.844 | 0.844 | 0.844 |
| **Aβ_40_** | ***β*** | 0.01 | 0.00 | 0.00 | 0.17 | 0.15 | -0.16 |
|  | ***Adj. P-value*** | 0.844 | 0.844 | 0.844 | 0.844 | 0.844 | 0.844 |
| **Aβ_42_** | ***β*** | -0.14 | 0.00 | 0.00 | 0.13 | 0.17 | -0.28 |
|  | ***Adj. P-value*** | 0.844 | 0.844 | 0.844 | 0.844 | 0.844 | 0.844 |
| **pTau-181/Aβ_42_** | ***β*** | 0.01 | 0.00 | 0.00 | -0.07 | 0.01 | 0.18 |
|  | ***Adj. P-value*** | 0.844 | 0.844 | 0.844 | 0.844 | 0.844 | 0.844 |
| **pTau-181** | ***β*** | -0.01 | 0.00 | 0.00 | 0.07 | 0.28 | 0.02 |
|  | ***Adj. P-value*** | 0.844 | 0.844 | 0.844 | 0.844 | 0.844 | 0.844 |
| **NfL** | ***β*** | -0.05 | 0.00 | 0.00 | 0.21 | 0.03 | 0.22 |
|  | ***Adj. P-value*** | 0.844 | 0.844 | 0.844 | 0.844 | 0.844 | 0.844 |
| **GFAP** | ***β*** | -0.04 | 0.00 | 0.00 | 0.16 | -0.05 | 0.06 |
|  | ***Adj. P-value*** | 0.844 | 0.844 | 0.844 | 0.844 | 0.844 | 0.844 |
| Abbreviations: Aβ, Amyloid-beta; GFAP, Glial Fibrillary Acidic Protein; LB, Lewy bodies; NfL, Neurofilament light chain; pTau, Phosphorylated tau; TDP-43, TAR DNA-binding protein 43. Values shown are the standardized beta coefficient (β) and the adjusted *P*-value from robust linear regression models. All models were adjusted for age at blood collection, sex, and the time interval between blood and brain donation. N=44-52. *P*-values were adjusted for false discovery rate (FDR) using the Benjamini & Hochberg method and scaled by the effective number of independent tests (Mₑff) estimated via the Li & Ji method; significance was set at Adj. *P* < 0.10. | | | | | | | |

| **Supplementary Table 6.**  **Robust linear regression associations between plasma biomarkers and measures of neuropathological substrates** | | | | | | | | | | | | |
| --- | --- | --- | --- | --- | --- | --- | --- | --- | --- | --- | --- | --- |
|  |  | **Thal Aβ phase** | **Braak NFT stage** | **CERAD NP score** | **ADNC score** | **Quantitative cortical total Aβ load** | **Quantitative cortical Aβ_40_ load** | **Quantitative cortical Aβ_42_ load** | **Quantitative cortical AT8 load** | **Quantitative cortical pTau-217 load** | **Quantitative cortical GT-38 load** | **Thal CAA stage** |
| **Aβ_42/40_ ratio** | ***β*** | -0.09 | 0.09 | 0.03 | -0.18 | -0.02 | -0.01 | -0.09 | 0.00 | 0.06 | 0.05 | -0.02 |
|  | ***Adj. P-value*** | 0.313 | 0.304 | 0.422 | 0.196 | 0.387 | 0.429 | 0.322 | 0.457 | 0.349 | 0.277 | 0.429 |
| **Aβ_40_** | ***β*** | -0.22 | -0.21 | -0.25 | -0.28 | -0.04 | -0.20 | -0.14 | 0.04 | 0.06 | 0.07 | -0.27 |
|  | ***Adj. P-value*** | 0.163 | 0.153 | 0.153 | 0.144 | 0.338 | 0.114 | 0.277 | 0.277 | 0.372 | 0.278 | 0.120 |
| **Aβ_42_** | ***β*** | -0.34 | -0.20 | -0.26 | -0.49 | -0.07 | -0.25 | -0.26 | 0.04 | 0.13 | 0.13 | -0.29 |
|  | ***Adj. P-value*** | **0.066** | 0.153 | 0.145 | **0.018** | 0.254 | **0.066** | 0.140 | 0.254 | 0.256 | 0.141 | 0.109 |
| **pTau-181/Aβ_42_** | ***β*** | 0.42 | 0.26 | 0.47 | 0.47 | 0.17 | 0.45 | 0.45 | 0.09 | 0.31 | 0.12 | 0.30 |
|  | ***Adj. P-value*** | **0.018** | **0.066** | **0.018** | **0.018** | **0.018** | **0.004** | **0.018** | **0.066** | **0.039** | 0.109 | **0.066** |
| **pTau-181** | ***β*** | 0.37 | 0.23 | 0.45 | 0.39 | 0.16 | 0.35 | 0.30 | 0.15 | 0.51 | 0.26 | 0.20 |
|  | ***Adj. P-value*** | **0.048** | 0.141 | **0.036** | **0.064** | **0.023** | **0.018** | **0.084** | **0.018** | **0.007** | **0.018** | 0.178 |
| **NfL** | ***β*** | 0.27 | 0.22 | 0.27 | 0.35 | 0.13 | 0.15 | 0.22 | 0.06 | 0.27 | 0.15 | 0.21 |
|  | ***Adj. P-value*** | 0.102 | 0.140 | 0.109 | **0.048** | **0.073** | 0.153 | 0.120 | **0.066** | **0.036** | **0.057** | 0.153 |
| **GFAP** | ***β*** | 0.06 | 0.08 | 0.07 | 0.20 | 0.12 | -0.01 | 0.10 | 0.02 | 0.12 | 0.05 | -0.04 |
|  | ***Adj. P-value*** | 0.377 | 0.347 | 0.372 | 0.211 | **0.097** | 0.429 | 0.313 | 0.313 | 0.250 | 0.295 | 0.402 |
| Abbreviations: Aβ - Amyloid-beta; ADNC - Alzheimer's Disease Neuropathologic Change; CERAD - Consortium to Establish a Registry for Alzheimer's Disease; Cortical AT8 load - Cortical phosphorylated Tau at positions 202/205 load; GFAP - Glial Fibrillary Acidic Protein; NfL - Neurofilament Light Chain; NFT - Neurofibrillary Tangle; NP - Neuritic Plaque; pTau-181 - Phosphorylated Tau at position 181. N=44-52. *P*-values were adjusted for false discovery rate (FDR) using the Benjamini & Hochberg method and scaled by the effective number of independent tests (Mₑff) estimated via the Li & Ji method; significance was set at Adj. *P* < 0.10. | | | | | | | | | | | | |

REFERENCES

1. Holstege H, Beker N, Dijkstra T, et al. The 100-plus Study of cognitively healthy centenarians: rationale, design and cohort description. *Eur J Epidemiol*. Dec 2018;33(12):1229-1249. doi:10.1007/s10654-018-0451-3

2. Tombaugh T, McDowell I, Kristjansson B, Hubley A. Mini-Mental State Examination (MMSE) and the Modified MMSE (3MS): a psychometric comparison and normative data. *Psychological Assessment*. 1996;8(1):48.

3. Folstein M, Folstein S, McHugh P. Mini-mental state”: a practical method for grading the cognitive state of patients for the clinician” J Psychiatr Res 12: 189–198. *Find this article online*. 1975;

4. Morris JC, Heyman A, Mohs RC, et al. The consortium to establish a registry for Alzheimer's disease (CERAD): I. Clinical and neuropsychological assessment of Alzheimer's disease. *Neurology*. 1989;

5. Lindeboom J, Schmand B, Tulner L, Walstra G, Jonker C. Visual association test to detect early dementia of the Alzheimer type. *Journal of Neurology, Neurosurgery & Psychiatry*. 2002;73(2):126-133.

6. Scale WDWAI. (WAIS-III). *San Antonio: The Psychological Corporation*. 1997;

7. Wilson BA, Evans JJ, Alderman N, Burgess PW, Emslie H. Behavioural assessment of the dysexecutive syndrome. *Methodology of frontal and executive function*. 1997;239:250.

8. Reitan R. Trail Making Test: Manual for administration, scoring and interpretation. *Bloomington: Indiana University*. 1956:134.

9. Munang L, Chan M, Lim W. Diagnostic performance of the clock drawing test using a pre-drawn circle in persons with early dementia. *Asian J Gerontol Geriatr*. 2010;5(2):54-61.

10. Shulman KI. Clock‐drawing: is it the ideal cognitive screening test? *International journal of geriatric psychiatry*. 2000;15(6):548-561.

11. Borkowski JG, Benton AL, Spreen O. Word fluency and brain damage. *Neuropsychologia*. 1967;5(2):135-140.

12. Kertesz A. *Western aphasia battery test manual*. Psychological Corporation; 1982.

13. Schmand B, Groenink S, Van den Dungen M. Letterfluency: psychometrische eigenschappen en Nederlandse normen. *Tijdschrift voor gerontologie en geriatrie*. 2008;39(2):64-74.

14. Yesavage JA, Sheikh JI. 9/Geriatric depression scale (GDS) recent evidence and development of a shorter version. *Clinical gerontologist*. 1986;5(1-2):165-173.

15. Mahoney FI. Functional evaluation: the Barthel index. *Maryland state medical journal*. 1965;14(2):61-65.

16. Jorm AF. The Informant Questionnaire on cognitive decline in the elderly (IQCODE): a review. *International psychogeriatrics*. 2004;16(3):275-293.

17. Thal DR, Rub U, Orantes M, Braak H. Phases of A beta-deposition in the human brain and its relevance for the development of AD. *Neurology*. Jun 25 2002;58(12):1791-800. doi:10.1212/wnl.58.12.1791

18. Braak H, Braak E. Neuropathological stageing of Alzheimer-related changes. *Acta neuropathologica*. 1991;82(4):239-59.

19. Mirra SS, Heyman A, McKeel D, et al. The Consortium to Establish a Registry for Alzheimer's Disease (CERAD). Part II. Standardization of the neuropathologic assessment of Alzheimer's disease. *Neurology*. Apr 1991;41(4):479-86. doi:10.1212/wnl.41.4.479

20. Thal DR, Ghebremedhin E, Orantes M, Wiestler OD. Vascular pathology in Alzheimer disease: correlation of cerebral amyloid angiopathy and arteriosclerosis/lipohyalinosis with cognitive decline. *J Neuropathol Exp Neurol*. Dec 2003;62(12):1287-301. doi:10.1093/jnen/62.12.1287

21. Nelson PT, Dickson DW, Trojanowski JQ, et al. Limbic-predominant age-related TDP-43 encephalopathy (LATE): consensus working group report. *Brain*. Jun 1 2019;142(6):1503-1527. doi:10.1093/brain/awz099

22. Bankhead P, Loughrey MB, Fernández JA, et al. QuPath: Open source software for digital pathology image analysis. *Sci Rep-Uk*. 2017;7(1):1-7.
